# Extracellular CIRP Induces Inflammation in Alveolar Type II Cells via TREM-1

**DOI:** 10.3389/fcell.2020.579157

**Published:** 2020-08-28

**Authors:** Chuyi Tan, Steven D. Gurien, William Royster, Monowar Aziz, Ping Wang

**Affiliations:** ^1^Center for Immunology and Inflammation, The Feinstein Institutes for Medical Research, Manhasset, NY, United States; ^2^Department of Surgery, Donald and Barbara Zucker School of Medicine at Hofstra/Northwell, Manhasset, NY, United States

**Keywords:** eCIRP, TREM-1, type II pneumocyte, inflammation, cytokine, chemokine

## Abstract

Extracellular cold-inducible RNA-binding protein (eCIRP) induces acute lung injury (ALI) in sepsis. Triggering receptor expressed on myeloid cells-1 (TREM-1) serves as a receptor for eCIRP to induce inflammation in macrophages and neutrophils. The effect of eCIRP on alveolar epithelial cells (AECs) remains unknown. We hypothesize that eCIRP induces inflammation in AECs through TREM-1. AECs were isolated from C57BL/6 mice and freshly isolated AECs were characterized as alveolar type II (ATII) cells by staining AECs with EpCAM, surfactant protein-C (SP-C), and T1 alpha (T1α) antibodies. AECs were stimulated with recombinant murine (rm) CIRP and assessed for TREM-1 by flow cytometry. ATII cells from WT and TREM-1^–/–^ mice were stimulated with rmCIRP and assessed for interleukin-6 (IL-6) and chemokine (C-X-C motif) ligand 2 (CXCL2) in the culture supernatants. ATII cells from WT mice were pretreated with vehicle (PBS), M3 (TREM-1 antagonist), and LP17 (TREM-1 antagonist) and then after stimulating the cells with rmCIRP, IL-6 and CXCL2 levels in the culture supernatants were assessed. All of the freshly isolated AECs were ATII cells as they expressed EpCAM and SP-C, but not T1α (ATI cells marker). Treatment of ATII cells with rmCIRP significantly increased TREM-1 expression by 56% compared to PBS-treated ATII cells. Stimulation of WT ATII cells with rmCIRP increased IL-6 and CXCL2 expression, while the expression of IL-6 and CXCL2 in TREM-1^–/–^ ATII cells were reduced by 14 and 23%, respectively. Pretreatment of ATII cells with M3 and LP17 significantly decreased the expression of IL-6 by 30 and 47%, respectively, and CXCL2 by 27 and 34%, respectively, compared to vehicle treated ATII cells after stimulation with rmCIRP. Thus, eCIRP induces inflammation in ATII cells via TREM-1 which implicates a novel pathophysiology of eCIRP-induced ALI and directs a possible therapeutic approach targeting eCIRP-TREM-1 interaction to attenuate ALI.

## Introduction

Acute lung injury (ALI) and acute respiratory distress syndrome (ARDS) are life-threatening complications of critically ill patients. They are characterized by severe inflammation, injury to the lungs, acute non-cardiogenic pulmonary edema, and hypoxemia (Ranieri et al., 2012). Pneumonia, sepsis, trauma, hemorrhage, and intestinal ischemia-reperfusion (I/R) often cause ALI (Matthay et al., 2019). The therapies of ALI are largely supportive and are often ineffective, leading to increased morbidity and mortality related to ALI (Bellani et al., 2016; Pham and Rubenfeld, 2017). Therefore, efforts focused on understanding the pathophysiology of ALI are important for finding new treatments.

Pathological specimens from patients with ALI and laboratory studies have demonstrated diffuse alveolar capillary barrier injury, increased permeability to liquids and proteins, and subsequent respiratory failure (Matthay et al., 2019). The alveolar capillary barrier is composed of squamous type I cells (ATI), cuboidal type II cells (ATII), interstitial space, and endothelium (Johnson and Matthay, 2010). The ATII cells secrete surfactant, a critical factor that reduces alveolar surface tension, allowing the alveoli to remain open, facilitating gas exchange (Ward and Nicholas, 1984). Injury to ATII cells results in decreased production of surfactant, which causes reduced lung compliance, leading to respiratory failure. The lung epithelium can be injured by pathogen-associated molecular patterns (PAMPs) such as bacterial products, viruses, and nucleic acids as well as damage-associated molecular patterns (DAMPs) which are endogenous danger molecules released by cells in states of stress such as hypoxia, mechanical force, sepsis, pancreatitis and other diseases (Saffarzadeh et al., 2012; Short et al., 2016; Matthay et al., 2019).

Cold-inducible RNA-binding protein (CIRP) is a glycine-rich RNA chaperone that facilitates RNA translation (Nishiyama et al., 1997). Upon release into the circulation, extracellular CIRP (eCIRP) serves as a DAMP which has a pro-inflammatory role in macrophages, neutrophils, lymphocytes, and endothelial cells (Aziz et al., 2019). In addition, increased expression of CIRP has been shown in the alveolar epithelium of lungs from chronic obstructive pulmonary disease (COPD) patients (Ran et al., 2016). The expression of CIRP in AECs was increased in mice treated with cold air (Chen et al., 2016). eCIRP’s role in activating lung macrophages and neutrophils has been identified, but its effects on alveolar epithelial cells remains unknown.

Triggering receptor expressed on myeloid cells-1 (TREM-1), an amplifier of inflammatory responses, is expressed on myeloid cells, such as neutrophils and monocytes (Colonna, 2003). The mRNA expression of TREM-1 is elevated in lung tissue of mice with ALI. This increased expression is related to the severity of the inflammatory response in ALI (Liu et al., 2010). Blocking TREM-1 has been shown to exhibit protective effects in lipopolysaccharide (LPS)-induced ALI via inhibiting the activation of the NLR family pyrin domain containing 3 (NLRP3) inflammasome (Liu et al., 2016). Although the pro-inflammatory effect of TREM-1 and its implication in the pathogenesis of ALI are emerging, the mechanisms remain poorly understood.

We have recently discovered that eCIRP is a new endogenous ligand of TREM-1 and that the binding of eCIRP to TREM-1 induces the production of cytokines in macrophages (Denning et al., 2020). TREM-1 expression in AECs at base line and after exposure to eCIRP remains unknown. Similarly, the direct effect of eCIRP on AECs is also unknown. Here, we hypothesize that eCIRP induces TREM-1 expression on AECs, leading to increased cytokine and chemokine release. In this study, we report that eCIRP induced the production of interleukin-6 (IL-6), chemokine (C-X-C motif) ligand 2 (CXCL2), and the expression of TREM-1 in ATII cells. Genetic depletion or pharmacological inhibition of TREM-1 decreased the production of IL-6 and CXCL2 in ATII cells. Thus, eCIRP activates AECs in a TREM-1-dependent manner and is a potential target for anti-inflammatory therapies.

## Materials and Methods

### Mice

C57BL/6 male mice were purchased from Charles River Laboratories (Wilmington, MA). TREM-1^–/–^ mice [Trem1tm1(KOMP)Vlcg] were generated by the trans-National Institutes of Health Knock-Out Mouse Project (KOMP) and obtained from the KOMP Repository University of California, Davis, CA. Age (8–12 weeks) matched healthy mice were used in all experiments. All mice were housed and kept at room temperature with normal chow and drinking water and housed individually with free access to food and water throughout the experiment. The mice were kept on a 12 h light/dark cycle. All animal experimental protocols were performed according to the guidelines on the use of experimental animals by The National Institutes of Health (Bethesda, MD). The protocol was approved by our Institutional Animal Care and Use Committees.

### Isolation of AECs

AECs were isolated from mice lungs as described previously (Chakraborty et al., 2017). In brief, mice were sacrificed by CO_2_ asphyxiation. Exsanguinated mice were made aseptic with ethanol spray, and a long ventral incision was made to expose the abdomen and chest cavity. The inferior vena cava was severed and the right heart was then perfused with cold PBS in order to flush the pulmonary vasculature. We then exposed the trachea, inserted a 22G shielded catheter into the lumen, and injected 2 ml of Dispase II (Sigma-Aldrich, St Louis, MO) through the trachea into the lungs. We instilled 0.5 ml of 1% liquefied agarose (Sigma-Aldrich) into the lungs. We then removed the lungs and placed them into 2 ml Dispase for 20 min at 37°C with constant rotation. After removing the lungs from the Dispase solution, we dissected the lung parenchyma using forceps in petri-dishes containing 7 ml of DMEM media supplemented with 1% glutamine, 1% penicillin/streptomycin, and 0.01% DNase I (Sigma-Aldrich). We filtered the cell suspension through 100, 70, 40, and 30 μm strainers (Corning Biosciences, Corning, NY). The filtrate was centrifuged for 15 min at 160 × g and treated with erythrocyte lysis buffer to eliminate the erythrocytes. The cell pellet was resuspended in 500 μl DMEM and incubated with biotinylated CD45 and CD16/32 antibodies (Biolegend, San Diego, CA) for 30 min. The cells were then incubated with streptavidin-coated magnetic beads for 30 min, and sorted by magnetic separation. The cells were then platted on petri-dishes for 4 h to remove adherent mesenchymal cells.

### Cell Culture

Freshly isolated AECs were plated in fibronectin-coated 48 well plates at a density of 1 × 10^5^ cells/well and cultured in airway epithelial cell growth medium along with the following supplements: bovine pituitary extract (0.004 ml/ml), epidermal growth factor (10 ng/ml), insulin (5 μg/ml), hydrocortisone (0.5 μg/ml), epinephrine (0.5 μg/ml), tri-iodo-l-thyronine (6.7 ng/ml), transferrin (10 μg/ml), and retinoic acid (0.1 ng/ml) all purchased from Promocell GmbH (Heidelberg, Germany). The cells were divided into two different treatment groups: AECs pre-treated with the TREM-1-eCIRP binding antagonist peptide M3 (RGFFRGG; GenScript USA Inc., Piscataway, NJ) (10 μg/ml) (Denning et al., 2020) or the TREM-1 decoy peptide LP17 (LQVTDSGLYRCVIYHPP; GenScript USA Inc.) (100 μg/ml) (Gibot et al., 2004). Both groups were pre-treated for 30 min, and then stimulated with recombinant mouse (rm) CIRP (1 μg/ml) for 24 h. The cells were not washed prior to the addition of rmCIRP. Then the supernatants were collected and stored at −20°C for cytokine and chemokine assays. rmCIRP was prepared in-house (Qiang et al., 2013). Briefly, rmCIRP was expressed in *E.coli*, and purified by using Ni^2+^-NTA column (Novagen, Madison, Wisconsin). The quality of the purified protein was assessed by Western blotting. The level of LPS in the purified protein was measured by a limulus amebocyte lysate (LAL) assay (Cambrex, East Rutherford, New Jersey). Only the purified protein lots that were endotoxin free were considered for *in vitro* and *in vivo* experiments.

### Assessment of TREM-1 Expression in AECs by Flow Cytometry

To detect TREM-1 expression in AECs, a total of 1 × 10^6^ AECs were plated in 6-well plates and then stimulated with PBS or rmCIRP (1 μg/ml) for 24 h. After the stimulation, the cells were washed with FACS buffer and stained with PE anti-mouse EpCAM antibody (clone: G8.8, Biolegend, San Diego, CA) and BV421 anti-mouse TREM-1 antibody (clone: 174031, BD Biosciences, San Jose, CA) for 30 min at 4°C. BV421 rat IgG2 antibody (clone: RTK2758, Biolegend) was used as an isotype Ab. Unstained cells were used to control flow cytometry’s voltage setting. Acquisition was performed on 30,000 events using a BD LSR Fortessa flow cytometer (BD Biosciences) and data were analyzed with FlowJo software (Tree Star, Ashland, OR).

### Immunofluorescent Staining

Immunofluorescent staining of freshly isolated AECs to determine their types was performed according to a protocol previously described (Chakraborty et al., 2017). In brief, AECs were platted on fibronectin-coated 8-well LabTek chambers for 1 or 7 days. The cells were washed once with cold PBS and fixed with 4% paraformaldehyde for 10 min at room temperature. The fixed cells were washed three times with PBS, followed by permeabilization by 0.1% Triton X-100 for 10 min. After washing the cells with PBS, they were blocked with 1% BSA for 1 h. Immunofluorescent staining was performed using primary antibodies against surfactant protein-C (SP-C) (Abcam, Cambridge, MA) and T1 alpha (T1α) (R&D Systems, Minneapolis, MN) and fluorescently tagged secondary antibodies. Primary antibodies were diluted in 1% BSA and incubated with the cells overnight at 4°C. After washing with PBS, cells were incubated with the second antibodies in 1% BSA for 1 h at room temperature in the dark. After an additional washing, slides were mounted immediately on Vectashield mounting medium with DAPI. The cells were visualized using fluorescent microscopy (Nikon BR, Tokyo, Japan).

### Enzyme-Linked Immunosorbent Assay

IL-6 and CXCL2 were measured in the culture supernatants of ATII cells following stimulation with rmCIRP by immunoreactivity in a double-sandwich enzyme-linked immunosorbent assay (ELISA) format using commercially available kits by following manufacturer’s instructions. The IL-6 ELISA kit was purchased from BD Biosciences and the CXCL2 ELISA kit was purchased from R&D Systems.

### Statistical Analysis

All statistical analyses were performed and the figures were prepared with GraphPad Prism version 7.0 software (GraphPad Software, La Jolla, CA). Comparisons between two groups were performed with a two-tailed Student’s *t-*test (parametric). Comparisons between multiple groups were analyzed using a one-way analysis of variance (ANOVA), followed by Student–Newman–Keuls (SNK) or Tukey’s multiple comparison test. The statistical significance was set at *p* < 0.05.

## Results

### Identification of Isolated Murine Alveolar Epithelial Cells

**FIGURE 1:**
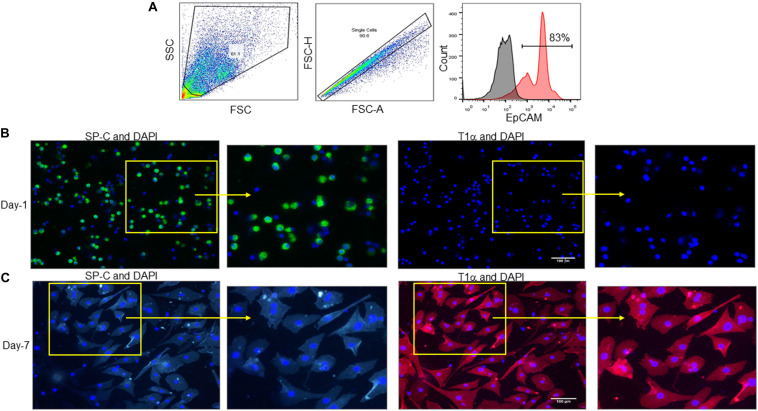
Identification of isolated murine alveolar epithelial cells. **(A)** AECs were isolated from C57BL/6 mice and stained with PE-EpCAM Ab, followed by fixing the cells and assessment by flow cytometry. **(A)** Representative dot blots and the histogram of the frequencies of EpCAM expressing cells are shown. Black histogram depicts isotype control, red histogram depicts EpCAM stained population. EpCAM, epithelial cell adhesion molecule. **(B,C)** AECs were isolated from C57BL/6 mice and cultured on fibronectin-coated culture plates for **(B)** 1 or **(C)** 7 days, then the cells were washed with PBS and stained with ATII cell specific marker SP-C (green) and ATI cell specific marker T1α (red) Abs. Nuclei were stained with DAPI (blue). Imaging was performed by fluorescent microscopy. Scale bars are 100 μm. Experiments were repeated at least two times, which generated reproducible findings.

A previously described protocol for isolation and culture of AECs was adopted to achieve the desired purification of AECs (Chakraborty et al., 2017). AECs were stained with antibodies against epithelial cell adhesion molecule (EpCAM), an epithelial cell-specific marker and analyzed by flow cytometry, which revealed the purity of sorted AECs to be 83% (Figure 1A). Our results were in agreement with the previous results of sorted AECs, which showed a purity of approximately 90% (Chakraborty et al., 2017). After isolation of primary murine AECs, all of the AECs were ATII cells, as characterized by their expression of SP-C, an ATII marker, but not T1α, an ATI marker (Figure 1B). To evaluate whether these cells were functionally active and capable of differentiation into ATI cells, the freshly isolated AECs (ATII) were cultured on fibronectin-coated culture plates for 7 days (Chakraborty et al., 2017). We found that after 7 days of culture of freshly isolated ATII cells, these cells differentiated into type I phenotype (ATI) as determined by their increased expression of T1α, but not SP-C (Figure 1C). Experiments were repeated at least two times, which generated reproducible findings. These data demonstrate that the freshly isolated AECs are mainly the ATII cells, which are viable and undergo differentiation into ATI cells.

### Stimulation of AECs With rmCIRP Induces the Production of IL-6 and CXCL2

**FIGURE 2:**
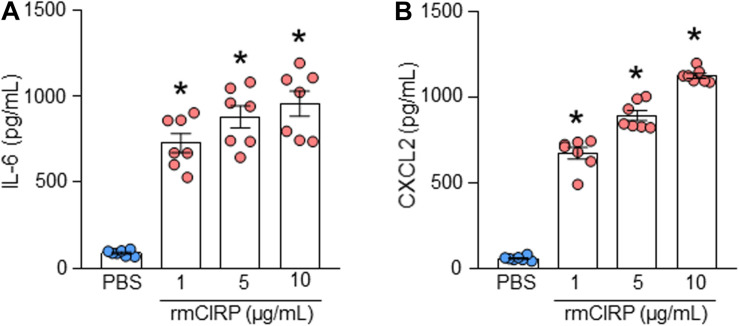
rmCIRP induces the production of IL-6 and CXCL2 in ATII cells. A total of 2 × 10^6^ AECs isolated from C57BL/6 mice were stimulated with PBS or rmCIRP (1, 5, 10 μg/ml) for 24 h. After stimulation, the supernatants of the cells were collected. The levels of **(A)** IL-6 and **(B)** CXCL2 in the supernatants were assessed by ELISA. Experiments were repeated at least three times using 3–4 samples/group each time. The figures represent the results of two experimental iterations combined together. We used 3–4 mice to isolate AECs, which usually gave rise to a total of 1.5–2 × 10^6^ AECs. Data are expressed as means ± SE (*n* = 7 samples/group). The groups were compared by one-way ANOVA and Tukey’s multiple comparison test (*p < 0.05 vs. PBS-treated group).

To determine the role of eCIRP on ATII cells, freshly isolated ATII cells were stimulated with increasing concentrations of rmCIRP. We found that ATII cells stimulated with rmCIRP significantly increased IL-6 production at doses of 1, 5, and 10 μg/ml, respectively, compared to PBS-treated cells (Figure 2A). Similarly, rmCIRP significantly increased the release of CXCL2 by AECII cells at doses of 1, 5, and 10 μg/ml, respectively, compared to PBS-treated cells (Figure 2B). The highest increase in the production of IL-6 and CXCL2 was found to occur at a dose of 10 μg/ml of rmCIRP. According to our previous studies (Denning et al., 2020; Murao et al., 2020), we chose 1 μg/ml of rmCIRP as an optimal stimulation concentration for the subsequent experiments. Therefore, eCIRP stimulation results in the release of pro-inflammatory cytokines and chemokines by alveolar epithelial type II cells in a dose-dependent manner.

### eCIRP Stimulation Increases the Expression of TREM-1 in ATII Cells

**FIGURE 3:**
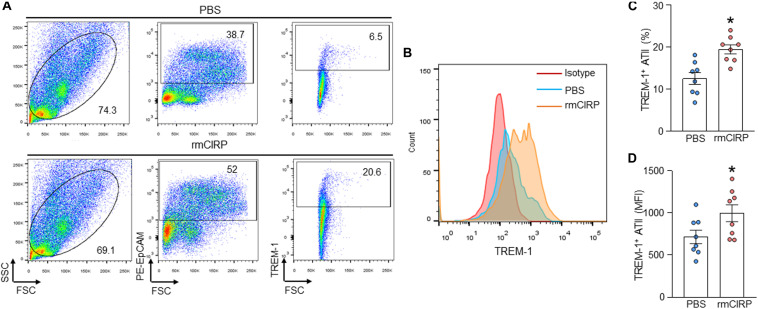
rmCIRP increases the expression of TREM-1 in ATII cells. **(A–D)** AECs (5 × 10^5^ cells) were isolated from C57BL/6 mice and were stimulated with PBS or rmCIRP (1 μg/ml) for 24 h. After stimulation, the cells were washed with PBS and stained with PE-EpCAM and BV421-TREM-1 Abs, followed by fixing the cells and assessment by flow cytometry. Representative **(A)** dot blots showing the gating strategy and **(C)** frequencies of TREM-1 expression and **(B)** histogram and **(D)** bar diagram showing mean immunofluorescence intensity (MFI) of TREM-1 expressing cells in EpCAM gated population are shown. Experiments were repeated at least three times, using 3–4 samples/group each time. The figures represent the results of two experimental iterations combined together. We used 3–4 mice to isolate AECs, which usually gave rise to a total of 1.5–2 × 10^6^ AECs. Data are expressed as means ± SE (*n* = 8 samples/group). The groups were compared by a two-tailed Student’s *t*-test (*p < 0.05 vs. PBS-treated group).

We previously identified eCIRP as a new ligand of TREM-1 in macrophages and neutrophils (Denning et al., 2020; Murao et al., 2020). The expression of TREM-1 and its role in eCIRP-mediated inflammation in AECs remain unknown. We assessed the expression of TREM-1 at the surface of AECs by flow cytometry after stimulation with rmCIRP. We found that under normal conditions, the TREM-1 expressing AEC population was minimal. However, after stimulation of AEC cells with rmCIRP, the frequency of TREM-1 expressing AECs was significantly increased by a mean value of 56% compared to PBS-treated AECs (Figures 3A,C). Akin to this result, we also found that after stimulation with rmCIRP the expression of TREM-1, in terms of MFI, was significantly increased by 39% compared to PBS-treated AEC cells (Figures 3B,D). Since all the freshly isolated pneumocytes were ATII (Figure 1), it suggests that following rmCIRP stimulation, TREM-1 expression was upregulated in ATII cells.

### TREM-1 Deficiency Results in Decreased Expression of IL-6 and CXCL2 in ATII Cells

**FIGURE 4:**
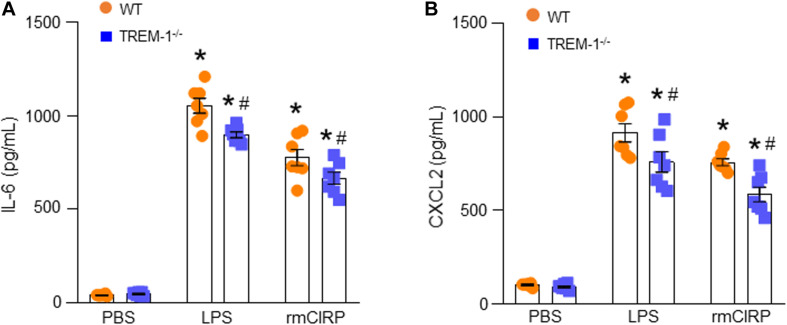
TREM-1 deficiency results in decreased expression of IL-6 and CXCL2 in ATII cells. A total of 2 × 10^6^ AECs isolated from WT or TREM-1^–/–^ mice were stimulated with PBS or rmCIRP (1 μg/ml) or LPS (100 ng/ml) for 24 h. After stimulation, the supernatants of the cells were collected. The levels of **(A)** IL-6 and **(B)** CXCL2 in the supernatants were assessed by ELISA. Experiments were repeated at least three times, using 3–4 samples/group each time. The figures represent the results of two experimental iterations combined together. We used 3–4 mice to isolate AECs, which usually gave rise to a total of 1.5–2 × 10^6^ AECs. Data are expressed as means ± SE (*n* = 7 samples/group). The groups were compared by one-way ANOVA and SNK method (*p < 0.05 vs. PBS-treated group, ^#^*p* < 0.05 vs. WT group).

We isolated AECs from WT and TREM-1^–/–^ mice, stimulated them with rmCIRP, and then assessed IL-6 and CXCL2 in the culture supernatant. We found that in both WT and TREM-1^–/–^ mice AECs, stimulation with rmCIRP significantly increased the expression of IL-6 and CXCL2 compared to PBS-treated cells isolated from WT and TREM-1^–/–^ mice (Figures 4A,B). We noticed that the production of IL-6 and CXCL2 were significantly decreased in rmCIRP-treated AECs isolated from TREM-1^–/–^ mice by 14 and 23%, respectively, compared to WT mice AECs (Figures 4A,B). Since TREM-1 acts as an amplifier of Toll-like receptor 4 (TLR4), we also focused on the effect of LPS induced expression of IL-6 and CXCL2 by AECs isolated from WT and TREM-1^–/–^ mice. We found that LPS stimulation of AECs from both WT and TREM-1^–/–^ mice significantly increased the expression of IL-6 and CXCL2. Nonetheless, we found significantly decreased expression of IL-6 and CXCL2 by 15 and 16% in AECs from TREM-1^–/–^ mice, compared to WT mice in response to LPS stimulation (Figures 4A,B). These data indicate that TREM-1 contributes to rmCIRP- and LPS-induced inflammation in AECs.

### Pharmacologic Inhibition of TREM-1 Attenuates IL-6 and CXCL2 Expression in ATII Cells

**FIGURE 5:**
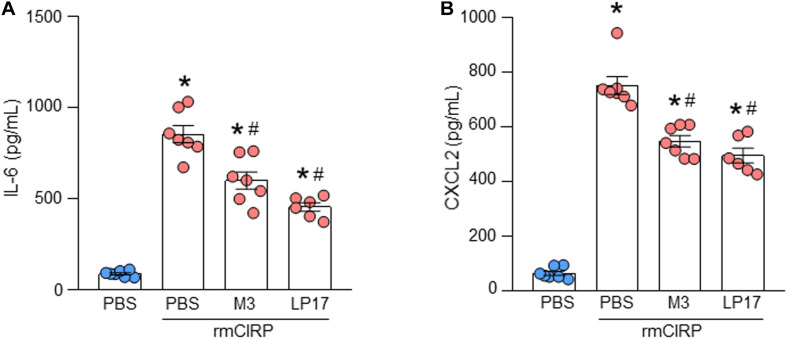
Pharmacologic inhibition of TREM-1 attenuates IL-6 and CXCL2 expression in ATII cells. A total of 2 × 10^6^ AECs isolated from C57BL/6 mice were treated with PBS, M3 (10 μg/ml), and LP17 (100 μg/ml). After 30 min of the pre-treatment the cells were then stimulated with rmCIRP at a dose of 1 μg/ml for 24 h. After stimulation, the culture supernatants were collected. The levels of **(A)** IL-6 and **(B)** CXCL2 in the cell culture supernatants were assessed by ELISA. Experiments were repeated at least three times, using 3–4 samples/group each time. The figures represent the results of two experimental iterations combined together. We used 3–4 mice to isolate AECs, which usually gave rise to a total of 1.5–2 × 10^6^ AECs. Data are expressed as means ± SE (*n* = 7 samples/group). The groups were compared by one-way ANOVA and SNK method (*p < 0.05 vs. PBS-treated group, ^#^*p* < 0.05 vs. rmCIRP-treated group).

To explore the role of TREM-1 in the activation of ATII cells, ATII cells were isolated from WT mice and cultured for 1 day. AECs were pre-treated with M3, an eCIRP-derived TREM-1 antagonist (Denning et al., 2020), and LP17, a TREM-1 decoy peptide (Gibot et al., 2004), for 30 min before stimulation with rmCIRP for 24 h. The supernatants were subsequently analyzed for IL-6 and CXCL2 contents by ELISA. We found that stimulation of AECs with rmCIRP significantly increased the expression of IL-6 and CXCL2 compared to PBS-treated cells (Figures 5A,B). On the other hand, the cells pre-treated with M3, and LP17 significantly decreased IL-6 expression by 30 and 47%, respectively, and CXCL2 expression by 27 and 34%, respectively, compared to vehicle (PBS) treatment in response to rmCIRP stimulation (Figures 5A,B). These data suggest that the pharmacologic inhibition of TREM-1 by M3 or LP-17 attenuates eCIRP-induced IL-6 and CXCL2 release in AEC cells.

## Discussion

eCIRP, a new DAMP, fuels inflammation by activating immune cells and parenchymal cells to produce pro-inflammatory cytokines, reactive oxygen species (ROS), and proteases. eCIRP subsequently promotes systemic inflammation and organ injury in various inflammatory diseases such as sepsis, hemorrhagic shock, ALI, and ischemia-reperfusion (I/R) injury (Qiang et al., 2013; Liu et al., 2016; Aziz et al., 2019). A recent study showed that eCIRP levels were up-regulated in the airway and alveolar epithelium of lungs from COPD patients (Ran et al., 2016). Intravenous injection of rmCIRP in healthy mice causes lung injury with evidence of increased leukocyte infiltration, enhanced production of pro-inflammatory cytokines, and vascular leakage and edema in the lung tissue (Yang et al., 2016). eCIRP induces lung injury by directly activating endothelial cells (ECs) and inducing EC pyroptosis (Yang et al., 2016). In addition, eCIRP causes sepsis-induced ALI by inducing endoplasmic reticulum (ER) stress and promoting downstream responses like apoptosis, NF-KB activation, and iNOS and pro-inflammatory cytokine production (Khan et al., 2017), while CIRP^–/–^ mice are protected from sepsis-induced ALI (Khan et al., 2017). Thus, eCIRP plays a critical role in the development of ALI.

Under infectious conditions, PAMPs, like LPS, are released into the alveoli and activate alveolar macrophages to release cytokines/chemokines and DAMPs, like eCIRP (Meduri et al., 1995; Qiang et al., 2013). DAMPs further cause alveolar capillary barrier injury, finally resulting in uncontrolled neutrophil infiltration and lung injury. AECs are an important part of the alveolar capillary barrier, which helps with gas exchange and protects the lungs from pathogens (Johnson and Matthay, 2010). Along with alveolar macrophages, alveolar epithelial cells are also the first cells to respond to PAMPs and DAMPs. Regulation of AECs response to these PAMPs and DAMPs is crucial to preserving the normal physiologic function of the alveolar-capillary barrier.

To study the effects of eCIRP on AECs, we isolated primary AECs from murine lungs, and stimulated the cells with rmCIRP. The freshly isolated AECs were mostly AEC type II cells. This is consistent with the previous study (Chakraborty et al., 2017). We found eCIRP significantly induced cytokine IL-6 and chemokine CXCL2 production in a dose dependent manner in ATII cells. These data indicate that eCIRP induces a pro-inflammatory phenotype in ATII cells. Some of the hallmark features of ALI are the increased infiltration of neutrophils in the lung tissues and elevated production of pro-inflammatory cytokines (Matthay et al., 2019). The migration of neutrophils requires the binding of chemokines to chemokine receptors. Interaction between CXCL2 and CXCR2 plays an important role in the recruitment of neutrophils into infection sites (Alves-Filho et al., 2009). In the present study, we found that eCIRP significantly increased chemokine CXCL2 expression in ATII cells. Our previous study showed that CIRP^–/–^ mice exhibited reduced lung injury with reduced infiltration of neutrophils in sepsis (Khan et al., 2017). This could be explained by the fact that sepsis induces eCIRP release into the lungs, which activates ATII cells to release cytokines and chemokines, such as IL-6 and CXCL2, resulting in a subsequent infiltration of neutrophils into the lung tissue causing ALI.

TREM-1 is predominantly expressed on myeloid cells such as macrophages and granulocytes (Bouchon et al., 2000). Prior studies have shown that during inflammation, TREM-1 is also detected on parenchymal cell types such as bronchial, gastric epithelial cells, and hepatic endothelial cells (Chen et al., 2008; Schmausser et al., 2008; Rigo et al., 2012; Tammaro et al., 2017). A previous study reported the mRNA and protein expression of TREM-1 in A549 cells, a human lung epithelial cell line (Liu et al., 2018). In the present study, our results show that murine resting alveolar epithelial cells have a low basal level of TREM-1 expression. TREM-1 is a potent amplifier of the inflammatory response and is associated with infectious diseases (Colonna, 2003). Recent evidence demonstrates that TREM-1 has a crucial role in the development of ALI and may be a potential therapeutic target for ALI and ARDS. The mRNA expression of TREM-1 was elevated in the lung tissue of mice with ALI. The elevated expression of TREM-1 was related to the severity of the inflammatory response in ALI (Liu et al., 2010). Blocking TREM-1 with LR12, a TREM-1 antagonist peptide, has shown a significant protective effect on LPS-induced acute lung injury via inhibiting the activation of the NLRP3 inflammasome (Liu et al., 2016).

We recently showed TREM-1 is a novel endogenous ligand of eCIRP and this interaction promotes an inflammatory response in sepsis (Denning et al., 2020). M3, a novel antagonist peptide of TREM-1, decreased eCIRP-induced systemic inflammation and tissue injury (Denning et al., 2020). This discovery led us to investigate the role of this receptor on the molecular mechanism underlying the activation of alveolar epithelial cells by eCIRP. In our current study, the results of flow cytometry showed that the expression of TREM-1 increased markedly in AECs after stimulation with eCIRP. M3 and LP17 are antagonists of TREM-1. M3 and LP17 suppressed the production of IL-6 and CXCL2 from eCIRP stimulated ATII cells, compared to PBS treated cells. In addition, IL-6 and CXCL2 release from LPS and eCIRP stimulated TREM-1^–/–^ ATII cells were lower than the ATII cells isolated from WT mice. The effect of inhibitors and gene knockout of TREM-1 results in an approximately 20% decrease in pro-inflammatory cytokine production by AECs. This can be explained by the fact that there are other signaling pathways involved in this effect. Toll-like receptor 4-myeloid differentiation factor 2 (TLR4-MD2) was expressed in low amounts on the resting respiratory epithelial cells, and LPS-induced activation of respiratory epithelial cells is dependent on the TLR4 signaling pathway (Guillot et al., 2004). Our previous study proved that eCIRP activates macrophages via its direct binding to the TLR4-MD2 complex (Qiang et al., 2013). In line with this finding, a recent study has revealed that S100A8, an alarmin activates alveolar epithelial cells in the context of acute lung injury in a TLR4-dependent manner (Chakraborty et al., 2017).

## Conclusion

In conclusion, our study revealed that resting respiratory epithelial cells express TREM-1 and that secretion of pro-inflammatory cytokine/chemokine upon exposure to eCIRP is a result of the TREM-1 signaling pathway. The discovery of the eCIRP/TREM-1 interaction involved in the activation of ATII cells will support the development of novel therapeutic targets for ALI or other lung diseases.

## Data Availability Statement

The raw data supporting the conclusions of this article will be made available by the authors, without undue reservation, to any qualified researcher.

## Ethics Statement

The animal study was reviewed and approved by the Feinstein Institutes for Medical Research Animal Care and Use Committees.

## Author Contributions

CT, SG, and MA designed the experiments. CT, SG, and WR performed the experiments. CT, MA, SG, and PW analyzed the data. MA and CT prepared the figures and wrote the manuscript. WR, SG, and PW reviewed and edited the draft. PW conceived the idea and supervised the project. All authors contributed to the article and approved the submitted version.

## Conflict of Interest

The authors declare that the research was conducted in the absence of any commercial or financial relationships that could be construed as a potential conflict of interest.

## Abbreviations

AECs: alveolar epithelial cells
ALI: acute lung injury
ATII: alveolar type II
CXCL2: chemokine (C-X-C motif) ligand 2
eCIRP: extracellular cold-inducible RNA-binding protein
EpCAM: epithelial cell adhesion molecule
IL-6: interleukin-6
NLRP3: NLR family pyrin domain containing 3
SP-C: surfactant protein-C
TLR4-MD2: Toll-like receptor 4-myeloid differentiation factor 2
TREM-1: triggering receptor expressed on myeloid cells-1.

## References

[B1] Alves-Filho J. C. Freitas A. Souto F. O. Spiller F. Paula-Neto H. Silva J. S. (2009). Regulation of chemokine receptor by Toll-like receptor 2 is critical to neutrophil migration and resistance to polymicrobial sepsis. *Proc. Natl. Acad. Sci. U.S.A.* 106 4018–4023. 10.1073/pnas.0900196106 19234125 PMC2656197

[B2] Aziz M. Brenner M. Wang P. (2019). Extracellular CIRP (eCIRP) and inflammation. *J. Leukoc. Biol.* 106 133–146. 10.1002/jlb.3mir1118-443r 30645013 PMC6597266

[B3] Bellani G. Laffey J. G. Pham T. Fan E. Brochard L. Esteban A. (2016). Epidemiology, patterns of care, and mortality for patients with acute respiratory distress syndrome in intensive care units in 50 Countries. *Jama* 315 788–800. 10.1001/jama.2016.0291 26903337

[B4] Bouchon A. Dietrich J. Colonna M. (2000). Cutting edge: inflammatory responses can be triggered by TREM-1, a novel receptor expressed on neutrophils and monocytes. *J. Immunol.* 164 4991–4995. 10.4049/jimmunol.164.10.4991 10799849

[B5] Chakraborty D. Zenker S. Rossaint J. Holscher A. Pohlen M. Zarbock A. (2017). Alarmin S100A8 activates alveolar epithelial cells in the context of acute lung injury in a TLR4-dependent manner. *Front. Immunol.* 8:1493. 10.3389/fimmu.2017.01493 29180999 PMC5693860

[B6] Chen L. Ran D. Xie W. Xu Q. Zhou X. (2016). Cold-inducible RNA-binding protein mediates cold air inducible airway mucin production through TLR4/NF-kappaB signaling pathway. *Int. Immunopharmacol.* 39 48–56. 10.1016/j.intimp.2016.07.007 27423012

[B7] Chen L. C. Laskin J. D. Gordon M. K. Laskin D. L. (2008). Regulation of TREM expression in hepatic macrophages and endothelial cells during acute endotoxemia. *Exp. Mol. Pathol.* 84 145–155. 10.1016/j.yexmp.2007.11.004 18222421 PMC2752215

[B8] Colonna M. (2003). TREMs in the immune system and beyond. *Nat. Rev. Immunol.* 3 445–453. 10.1038/nri1106 12776204

[B9] Denning N. L. Aziz M. Murao A. Gurien S. D. Ochani M. Prince J. M. (2020). Extracellular CIRP as an endogenous TREM-1 ligand to fuel inflammation in sepsis. *JCI Insight* 5:e134172. 10.1172/jci.insight.134172 32027618 PMC7141396

[B10] Gibot S. Kolopp-Sarda M. N. Béné M. C. Bollaert P. E. Lozniewski A. Mory F. (2004). A soluble form of the triggering receptor expressed on myeloid cells-1 modulates the inflammatory response in murine sepsis. *J. Exp. Med.* 200 1419–1426. 10.1084/jem.20040708 15557347 PMC2211948

[B11] Guillot L. Medjane S. Le-Barillec K. Balloy V. Danel C. Chignard M. (2004). Response of human pulmonary epithelial cells to lipopolysaccharide involves Toll-like receptor 4 (TLR4)-dependent signaling pathways: evidence for an intracellular compartmentalization of TLR4. *J. Biol. Chem.* 279 2712–2718. 10.1074/jbc.M305790200 14600154

[B12] Johnson E. R. Matthay M. A. (2010). Acute lung injury: epidemiology, pathogenesis, and treatment. *J. Aerosol. Med. Pulm. Drug. Deliv.* 23 243–252. 10.1089/jamp.2009.0775 20073554 PMC3133560

[B13] Khan M. M. Yang W. L. Brenner M. Bolognese A. C. Wang P. (2017). Cold-inducible RNA-binding protein (CIRP) causes sepsis-associated acute lung injury via induction of endoplasmic reticulum stress. *Sci. Rep.* 7:41363. 10.1038/srep41363 28128330 PMC5269663

[B14] Liu F. Zhang X. Zhang B. Mao W. Liu T. Sun M. (2018). TREM1: a positive regulator for inflammatory response via NF-κB pathway in A549 cells infected with Mycoplasma pneumoniae. *Biomed. Pharmacother.* 107 1466–1472. 10.1016/j.biopha.2018.07.176 30257363

[B15] Liu N. Gu Q. Zheng Y. S. (2010). Expression of triggering receptor-1 in myeloid cells of mice with acute lung injury. *World J. Emerg. Med.* 1 144–148. 25214958 PMC4129757

[B16] Liu T. Zhou Y. Li P. Duan J. X. Liu Y. P. Sun G. Y. (2016). Blocking triggering receptor expressed on myeloid cells-1 attenuates lipopolysaccharide-induced acute lung injury via inhibiting NLRP3 inflammasome activation. *Sci. Rep.* 6:39473. 10.1038/srep39473 28004759 PMC5177963

[B17] Matthay M. A. Zemans R. L. Zimmerman G. A. Arabi Y. M. Beitler J. R. Mercat A. (2019). Acute respiratory distress syndrome. *Nat. Rev. Dis. Primers* 5:18. 10.1038/s41572-019-0069-0 30872586 PMC6709677

[B18] Meduri G. U. Kohler G. Headley S. Tolley E. Stentz F. Postlethwaite A. (1995). Inflammatory cytokines in the BAL of patients with ARDS. Persistent elevation over time predicts poor outcome. *Chest* 108 1303–1314. 10.1378/chest.108.5.1303 7587434

[B19] Murao A. Arif A. Brenner M. Denning N. L. Jin H. Takizawa S. (2020). Extracellular CIRP and TREM-1 axis promotes ICAM-1-Rho-mediated NETosis in sepsis. *Faseb J.* 34 9771–9786. 10.1096/fj.202000482R 32506691 PMC9092350

[B20] Nishiyama H. Itoh K. Kaneko Y. Kishishita M. Yoshida O. Fujita J. (1997). A glycine-rich RNA-binding protein mediating cold-inducible suppression of mammalian cell growth. *J. Cell Biol.* 137 899–908. 10.1083/jcb.137.4.899 9151692 PMC2139845

[B21] Pham T. Rubenfeld G. D. (2017). Fifty years of research in ARDS. the epidemiology of acute respiratory distress syndrome. a 50th birthday review. *Am. J. Respir. Crit. Care Med.* 195 860–870. 10.1164/rccm.201609-1773CP 28157386

[B22] Qiang X. Yang W. L. Wu R. Zhou M. Jacob A. Dong W. (2013). Cold-inducible RNA-binding protein (CIRP) triggers inflammatory responses in hemorrhagic shock and sepsis. *Nat. Med.* 19 1489–1495. 10.1038/nm.3368 24097189 PMC3826915

[B23] Ran D. Chen L. Xie W. Xu Q. Han Z. Huang H. (2016). Cold-inducible RNA binding protein regulates mucin expression induced by cold temperatures in human airway epithelial cells. *Arch. Biochem. Biophys.* 603 81–90. 10.1016/j.abb.2016.05.009 27184164

[B24] Ranieri V. M. Rubenfeld G. D. Thompson B. T. Ferguson N. D. Caldwell E. Fan E. (2012). Acute respiratory distress syndrome: the Berlin Definition. *Jama* 307 2526–2533. 10.1001/jama.2012.5669 22797452

[B25] Rigo I. McMahon L. Dhawan P. Christakos S. Yim S. Ryan L. K. (2012). Induction of triggering receptor expressed on myeloid cells (TREM-1) in airway epithelial cells by 1,25(OH)2 vitamin D3. *Innate Immun.* 18 250–257. 10.1177/1753425911399796 21690199 PMC3179813

[B26] Saffarzadeh M. Juenemann C. Queisser M. A. Lochnit G. Barreto G. Galuska S. P. (2012). Neutrophil extracellular traps directly induce epithelial and endothelial cell death: a predominant role of histones. *PLoS One* 7:e32366. 10.1371/journal.pone.0032366 22389696 PMC3289648

[B27] Schmausser B. Endrich S. Beier D. Moran A. P. Burek C. J. Rosenwald A. (2008). Triggering receptor expressed on myeloid cells-1 (TREM-1) expression on gastric epithelium: implication for a role of TREM-1 in *Helicobacter* pylori infection. *Clin. Exp. Immunol.* 152 88–94. 10.1111/j.1365-2249.2008.03608.x 18321350 PMC2384064

[B28] Short K. R. Kasper J. van der Aa S. Andeweg A. C. Zaaraoui-Boutahar F. Goeijenbier M. (2016). Influenza virus damages the alveolar barrier by disrupting epithelial cell tight junctions. *Eur. Respir. J.* 47 954–966. 10.1183/13993003.01282-2015 26743480

[B29] Tammaro A. Derive M. Gibot S. Leemans J. C. Florquin S. Dessing M. C. (2017). TREM-1 and its potential ligands in non-infectious diseases: from biology to clinical perspectives. *Pharmacol. Ther.* 177 81–95. 10.1016/j.pharmthera.2017.02.043 28245991

[B30] Ward H. E. Nicholas T. E. (1984). Alveolar type I and type II cells. *Aust. N. Z. J. Med.* 14 (5 Suppl. 3), 731–734. 10.1111/j.1445-5994.1984.tb04343.x 6598039

[B31] Yang W. L. Sharma A. Wang Z. Li Z. Fan J. Wang P. (2016). Cold-inducible RNA-binding protein causes endothelial dysfunction via activation of Nlrp3 inflammasome. *Sci. Rep.* 6:26571. 10.1038/srep26571 27217302 PMC4877585

